# Enhanced Local Skeletal Muscle Oxidative Capacity and Microvascular Blood Flow Following 7-Day Ischemic Preconditioning in Healthy Humans

**DOI:** 10.3389/fphys.2018.00463

**Published:** 2018-05-09

**Authors:** Owen Jeffries, Mark Waldron, John R. Pattison, Stephen D. Patterson

**Affiliations:** ^1^School of Sport, Health and Applied Science, St Mary’s University, London, United Kingdom; ^2^School of Biomedical Science, Newcastle University, Newcastle upon Tyne, United Kingdom; ^3^School of Science and Technology, University of New England, Armidale, NSW, Australia

**Keywords:** NIRS, blood flow restriction, exercise, ischemia, mitochondria

## Abstract

Ischemic preconditioning (IPC), which involves intermittent periods of ischemia followed by reperfusion, is an effective clinical intervention that reduces the risk of myocardial injury and confers ischemic tolerance to skeletal muscle. Repeated bouts of IPC have been shown to stimulate long-term changes vascular function, however, it is unclear what metabolic adaptations may occur locally in the muscle. Therefore, we investigated 7 days of bilateral lower limb IPC (4 × 5 min) above limb occlusion pressure (220 mmHg; *n* = 10), or sham (20 mmHg; *n* = 10), on local muscle oxidative capacity and microvascular blood flow. Oxidative capacity was measured using near-infrared spectroscopy (NIRS) during repeated short duration arterial occlusions (300 mmHg). Microvascular blood flow was assessed during the recovery from submaximal isometric plantar flexion exercises at 40 and 60% of maximal voluntary contraction (MVC). Following the intervention period, beyond the late phase of protection (72 h), muscle oxidative recovery kinetics were speeded by 13% (rate constant pre 2.89 ± 0.47 min^-1^ vs. post 3.32 ± 0.69 min^-1^; *P* < 0.05) and resting muscle oxygen consumption (m
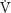
O_2_) was reduced by 16.4% (pre 0.39 ± 0.16%.s^-1^ vs. post 0.33 ± 0.14%.s^-1^; *P* < 0.05). During exercise, changes in deoxygenated hemoglobin (HHb) from rest to steady state were reduced at 40 and 60% MVC (16 and 12%, respectively, *P* < 0.05) despite similar measures of total hemoglobin (tHb). At the cessation of exercise, the time constant for recovery in oxygenated hemoglobin (O_2_Hb) was accelerated at 40 and 60% MVC (by 33 and 43%, respectively) suggesting enhanced reoxygenation in the muscle. No changes were reported for systemic measures of resting heart rate or blood pressure. In conclusion, repeated bouts of IPC over 7 consecutive days increased skeletal muscle oxidative capacity and microvascular muscle blood flow. These findings are consistent with enhanced mitochondrial and vascular function following repeated IPC and may be of clinical or sporting interest to enhance or offset reductions in muscle oxidative capacity.

## Introduction

Ischemic preconditioning (IPC), first described by Murry et al. (1986), is a technique that intermittently occludes circulatory blood flow, interspersed by periods of tissue reperfusion. Applied in this way, local IPC protects the myocardium from tissue injury following ischemic insult (Murry et al., 1986). In addition, remote adaptations in distal tissues not directly exposed to the ischemic stimulus have also been observed (Hausenloy and Yellon, 2008). Since its early inception, IPC has been used for many different purposes and can be applied acutely or repeated across a number of days. Acute IPC has been shown to protect organs, including the myocardium (Murry et al., 1986), kidney (Lee and Emala, 2000), liver (Yoshizumi et al., 1998), and skeletal muscle (Gurke et al., 2000), from the damage caused by a subsequent prolonged ischemic event. In addition, systemic effects of IPC have also been reported to modulate parasympathetic nervous system activity (Enko et al., 2011). Furthermore, an acute application of IPC has beneficial effects on exercise performance (de Groot et al., 2010; Kido et al., 2015; Patterson et al., 2015; Tanaka et al., 2016). Protection conferred from an acute exposure of IPC has led investigators to examine whether repeated application may elicit dose-dependent protection. Indeed, repeated IPC has been shown to offer greater protection against cardiac ischemia, in a dose-dependent manor, in animal models (Wei et al., 2011; Yamaguchi et al., 2015). In humans, repeated IPC has been applied across 7 consecutive days (Jones et al., 2014), episodically across an 8-week period (Jones et al., 2015), and twice daily application for 300 days (Meng et al., 2012). Here clinical efficacy has been shown in patients displaying reduced stroke recurrence (Meng et al., 2012), improved wound healing in diabetics (Shaked et al., 2015), increased coronary flow reserve in heart failure patients (Kono et al., 2014), reductions in systolic and diastolic blood pressure (Madias, 2011; Jones et al., 2014), and improved vascular health (Kimura et al., 2007; Jones et al., 2014, 2015). However, the mechanisms by which IPC exerts protection are not well understood. Two phases of protection have been described were an early phase offers immediate protection which lasts several hours, linked to the release of mediators such a bradykinin and adenosine. A second window of protection, the late phase, follows approximately 24 h later lasting between 48 and 72 h and is dependent on the induction of protective proteins (Loukogeorgakis et al., 2005).

Acute application of IPC has also been shown to improve local skeletal muscle oxygenation during exercise (Saito et al., 2004; Kido et al., 2015; Patterson et al., 2015; Tanaka et al., 2016). For example, Patterson et al. (2015) demonstrated an improved maintenance of muscle oxygenation during repeated sprint cycling during the early phase of protection. Furthermore, IPC enhances muscle deoxygenation dynamics during whole-body (Kido et al., 2015) and local muscular endurance exercise (Tanaka et al., 2016), suggestive of lower O_2_ extraction and/or greater blood flow to skeletal muscle. In skeletal muscle, local application of IPC confers ischemic tolerance to subsequent ischemic events (Gurke et al., 2000). There is also evidence that IPC can protect against reductions in ischemic-induced glycogen depletion (Lintz et al., 2013), restore mitochondrial dysfunction (Thaveau et al., 2007; Mansour et al., 2012) and remotely lower energy metabolism during sustained ischemia (Addison et al., 2003), thus demonstrating a range of potential mechanisms whereby skeletal muscle function is enhanced. However, to date, little is known about the oxidative potential of skeletal muscle following repeated bouts of IPC.

The energetic demands of muscular work are largely supported by oxidative metabolism. Using near-infrared spectroscopy (NIRS), muscle oxidative capacity is reduced in clinical patient groups where normal muscle function is impaired, such as in motor-complete spinal cord injuries (Erickson et al., 2013), peripheral vascular disorders (Cheatle et al., 1991), or those with sedentary lifestyles (Brizendine et al., 2013). In comparison, endurance trained athletes display 5-fold increases in muscle oxidative function (Brizendine et al., 2013; Adami and Rossiter, 2017). Using NIRS alongside a series of transient arterial occlusions has enabled non-invasive reporting of muscle oxidative capacity with good reproducibility when compared to gold standard techniques such as magnetic resonance spectroscopy (MRS) (Ryan et al., 2013c) and *in situ* measures of respiratory capacity via muscle biopsy analysis (Ryan et al., 2014). Interventions that can stimulate adaptations in skeletal muscle function, other than physical exercise, may not only be useful for athletic populations but could improve or sustain physical activity in clinical populations, or facilitate recovery following brief periods of immobility. The reported increases in vascular conductance following repeated IPC (Jones et al., 2014), alongside demonstrable effects of acute IPC on exercise performance and local muscle O_2_ dynamics (Kido et al., 2015; Patterson et al., 2015), led us to hypothesize that repeated bouts of IPC may stimulate a sustained enhancement of skeletal muscle oxidative capacity and microvascular blood flow. Therefore, we examined the effects of 7 consecutive days of bilateral lower-limb IPC on local skeletal muscle oxidative capacity and microvascular blood flow in healthy, young men.

## Materials and Methods

### Participants

A total of 20 adult male participants volunteered for this study (age, 26 ± 5 years; stature, 180 ± 6 cm; body mass 80 ± 12 kg). The participants were all actively engaged in regular exercise (250 ± 150 min/week) that consisted of endurance running or where involved in team sports. They were non-smokers and not taking any medications. Participants were advised to maintain a normal training schedule throughout the IPC or sham intervention but not to undertake any additional training or to reduce their current training load. *A priori* sample size was calculated using G^∗^Power (Version 3.1.9.3). This was determined according to changes in the deoxygenated hemoglobin/myoglobin (HHb) time course for recovery from moderate-intensity exercise following acute application of IPC [Δtime constant (Tc) = 4.6 s; *SD* = 1.7] (Kido et al., 2015). A total of 6 participants per group were deemed sufficient to yield a power of 0.80 and α = 0.05. A total of 10 participants were recruited to account for experimental mortality throughout the duration of the trial. Ethical approval was provided by the St. Mary’s University Ethics Committee which was conducted in accordance with the 1964 Helsinki Declaration. All participants provided written, informed consent before testing.

### Experimental Design

Participants first visited the laboratory to provide informed consent, conduct a maximal voluntary plantar flexion contraction (MVC) for determination of subsequent exercise intensities and for the purposes of allocating groups. Participants were also familiarized with the IPC pressure and electrical stimulation procedures used in the experimental trials. The main experimental trial began 48–72 h later after which participants attended the laboratory on nine occasions, across 11 consecutive days. All trials were conducted at the same time of day to eliminate circadian variation. On the first experimental visit, baseline measurements of muscle oxygenation were performed, followed by a series of arterial occlusions to establish resting muscle oxygen consumption (m
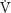
O_2_) and the rate of recovery from a brief electrical stimulation. An exercise protocol involving plantar flexion isometric contractions over a range of submaximal exercise intensities was performed on a dynamometer. Participants were ranked based on their MVC performance during the first visit and separated into an intervention (IPC) or sham (control) group using A-B-B-A. Each group then visited the laboratory on 7 consecutive days to perform an IPC or sham procedure. After the final intervention, participants returned to the laboratory 72 h later to replicate the tests performed on the first experimental visit. The 72-h time period was chosen as this was outside the reported early and late phases of protection conferred by IPC (Loukogeorgakis et al., 2005) and therefore would reflect a sustained adaptation to the IPC stimulus. Participants were instructed to avoid consumption of alcohol or caffeinated products for 24 h before, and strenuous exercise 48 h before, the baseline and post-intervention test.

### Measures of m$\overset{˙}{V}$O_2_

**FIGURE 1:**
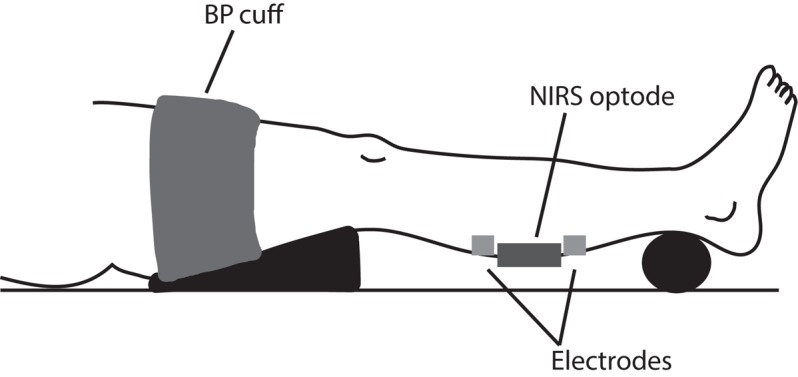
Experimental setup.

Following 10 min of supine rest on a padded table with both legs fully extended, a triangular pillow was placed under the right thigh and support placed under the participant’s right ankle to optimize the lower leg position perpendicular to the floor (**Figure 1**). Participants were informed to not move their limbs throughout the testing procedures. A near-infrared spectroscopy (NIRS) optode (Portamon, Artinis medical systems) was placed on the gastrocnemius medialis (2/3 distance from the calcaneus and anterior fossa) and secured with an elastic bandage (Tiger Tear, Hampshire, United Kingdom) to prevent movement and covered with an optically dense black material to minimize the intrusion of extraneous light. The position of the probe was marked with indelible ink which was reapplied at regular intervals during the intervention protocol to ensure correct placement of the optode during the post-intervention trial. Two square electrodes (50 × 50 mm), attached to a neuromuscular stimulator (NeuroTrac Sports, Verity Medical LTD, Hampshire, United Kingdom), were placed immediately next to the NIRS optode at proximal and distal skin sites. A rapid inflating blood pressure cuff (width: 15 cm; Hokanson SC12D, Bellevue, WA, United States) in conjunction with a rapid cuff inflator (E20, Hokanson, Bellevue, WA, United States) supplied by an air compressor, was placed around the upper thigh.

**FIGURE 2:**
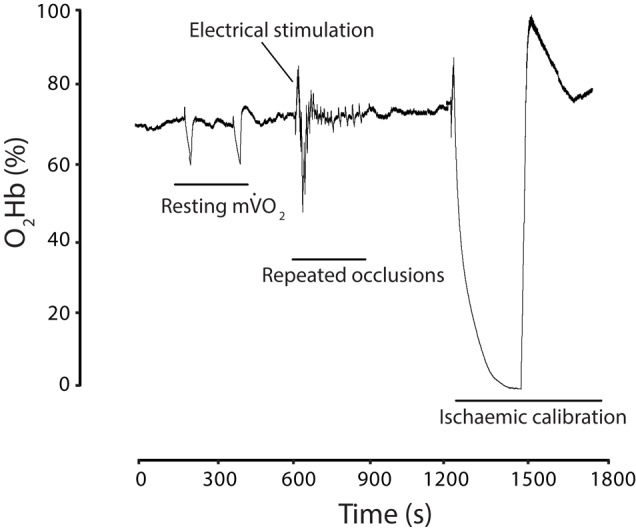
Experimental trial illustrated with muscle oxygenated hemoglobin (O_2_Hb) expressed as a percentage of an ischemic normalization. Two resting 30-s occlusions were performed to establish resting m
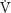
O_2_, followed by a 15-s electrical stimulation and a series of 15 brief occlusions. This was repeated but not shown for clarity. Finally, a 5-min ischemic normalization procedure was performed.

Resting m
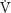
O_2_ was assessed by rapidly inflating the blood pressure cuff to 300 mmHg, ensuring full arterial and venous occlusion of the lower limb for 30 s. This procedure was then repeated after 3-min rest, with data averaged over the two measures. Following a further 3-min rest, the main test protocol began. To examine muscle oxidative recovery, twitch electrical stimulation (pulse duration 250 μS) was administered at a frequency of 6 Hz (Ryan et al., 2013a) for 15 s to increase muscle metabolic rate. Electromyostimulation allows non-selective and synchronous recruitment of muscle fibers in the region of interest (Gregory and Bickel, 2005). Current intensity was gradually increased over an initial 10 s to 60 mA for patient comfort (Doucet et al., 2012). The chosen current intensity elicited a twitch contraction across all individuals tested. It has previously been noted that small differences in stimulation do not influence measurements of m
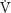
O_2_ recovery kinetics (Ryan et al., 2013a). Immediately following the stimulation, a series of brief arterial occlusions (300 mmHg) were performed using the following protocol: cuff 1–5 = 5 s ON, 5 s OFF; cuff 6–10 = 5 s ON, 10 s OFF; cuff 11–15 = 10 s ON, 20 s OFF (Ryan et al., 2012). This protocol was used to characterize the recovery of m
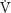
O_2_ and has been shown to be reliable and reproducible when compared to phosphorus magnetic resonance spectroscopy (P-MRS) indexes of skeletal muscle oxidative function (Ryan et al., 2013c), and *in situ* high-resolution respirometry measures of mitochondrial respiratory capacity via muscle biopsy analysis (Ryan et al., 2014). The exercise/cuff protocol was performed twice with a 5-min rest period between protocols and the two tests were then averaged. To normalize the NIRS signal, an ischemia/hyperemia procedure was performed as follows. Following a brief 5-s electrical stimulation at 6 Hz, the cuff was inflated to 300 mmHg for 5 min (or until a plateau was reached). This represented maximal deoxygenation (0%) of the tissue under the optode. Following release of the cuff, a peak hyperemic response (representing 100% oxygenation) was recorded. The short duration stimulation helped to minimize the duration of the ischemic cuff and avoid unnecessary discomfort. All data were normalized within each trial to this ‘physiological’ range to allow comparisons between individuals with differing ATT (Van Beekvelt et al., 2001; Ryan et al., 2012). Representative experimental data from a single individual trial is shown (**Figure 2**).

### Calculation of m$\overset{˙}{V}$O_2_

A blood volume correction factor was applied to each data point as previously described (Ryan et al., 2012) based on the assumption that during an arterial occlusion, the area under the probe is a closed system and therefore changes in oxygenated hemoglobin/myoglobin (O_2_Hb) and deoxygenated hemoglobin/myoglobin (HHb) should occur in a 1:1 ratio. Briefly, a blood volume correction factor was calculated according to Eq. 1:

(1) $$\beta (t)=\frac{\left[{\text{O}}_{2}\text{Hb}(t)\right]}{\left(O_{2}\text{Hb})+[\text{HHb}(t)\right]}$$

where, β is the blood volume correction factor, *t* represents time, O_2_Hb is the oxygenated hemoglobin/myoglobin signal and HHb is the deoxygenated hemoglobin/myoglobin signal. The blood volume correction factor was calculated for each data point and then applied to the raw NIRS data for O_2_Hb (Eq. 2) and HHb (Eq. 3) as below:

(2) $${\text{O}}_{2}{\text{Hb}}_{\text{c}}(t)={\text{O}}_{2}\text{Hb}(t)-[t\text{Hb}(t)\times (1-\beta )]$$

(3) $${\text{HHb}}_{\text{c}}(t)=\text{HHb}(t)-[t\text{Hb(t)}\times \beta ]$$

where, O_2_Hb_c_ and HHb_c_ are the corrected oxygenated and deoxygenated hemoglobin/myoglobin signals, tHb is the relative total hemoglobin concentration from the NIRS device, β is the blood volume correction factor, and *t* is time. These values for O_2_Hb_c_ and HHb_c_ thus represent the corrected data minus any blood volume changes.

The m
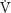
O_2_ was calculated by the slope of change in the corrected O_2_Hb and HHb during the first 3 s of an arterial occlusion by using simple linear regression. We calculated a test–re-test reliability of 6.7% coefficient of variation (CV) for resting m
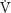
O_2._

Following an electrically stimulated increase in metabolic rate, the repeated m
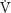
O_2_ measurements were then fitted to a monoexponential curve to derive a rate constant (*k*). This method utilizes linear modeling to report changes in m
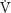
O_2_ as an index of oxidative capacity (Hamaoka et al., 1996), according to Eq. 4:

(4) $$y=End-Delta\times (1=e^{-kt})$$

where *y* is the relative m
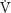
O_2_ during arterial occlusion, End is the m
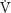
O_2_ immediately following the cessation of the electrical stimulation, Delta is the change in m
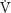
O_2_ from rest to end exercise, *t* is time, and *k* is the rate constant. The recovery of m
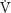
O_2_ to resting levels, therefore, represents the maximal oxidative capacity of the region of interest monitored in skeletal muscle. We calculated a test–re-test reliability for the *k* assessment of oxidative function using NIRS as 8.7% CV, which was comparable to that reported elsewhere for the same technique (Adami and Rossiter, 2017).

### Submaximal Plantar Flexion Exercise

A total of 14 participants from the initial study (age, 26 ± 5 years; stature, 182 ± 5 cm; body mass 84 ± 12 kg) took part in an exercise protocol to further identify changes in local muscle metabolism and blood flow. Seven participants per group were recruited to complete this follow-up investigation due to availability of laboratory and the duration of the expanded testing protocol required. Participants (IPC *n* = 7; Sham *n* = 7) conducted a range submaximal isometric plantar flexion exercise tasks following tests of muscle oxidative capacity. They were seated upright on a dynamometer [Kin Com Auto Positioning (125 AP), Chattanooga Group Inc., Hixson, TN, United States] with the knee fully extended (0^o^) to ensure that the gastrocnemius contributed significantly to plantar flexor joint movement (Cresswell et al., 1995) and the hip joint was extended (124 ± 4^o^), as assessed using a goniometer. The foot was fixed in a neutral anatomical position, with the sole of the foot fixed perpendicular (90^o^) to the tibia. The lateral malleolus was visually aligned with the center of rotation of the dynamometer lever arm and the foot was securely fixed to the dynamometer foot plate with Velcro straps. All measures for each participant were recorded for future trials. Participants conducted a warm-up, comprising of 3 × 5-s submaximal isometric plantar flexion contractions at 25, 50, and 75% of perceived maximum effort, separated by 30-s rest. Maximal voluntary plantar flexion isometric contraction (MVC) was first established during the familiarization session to enable determination of a range of working submaximal exercise intensities for pre and post exercise testing. Participants were instructed to perform three maximal isometric plantar flexion contractions and gradually develop force from rest to maximum over a 3- to 5-s time period, separated by 60-s rest. The peak plantar flexion torque associated with maximal voluntary contraction (MVC) was then recorded. If the 3rd contraction continued to increase, additional attempts were made until a plateau was reached.

During experimental visits participants performed intermittent plantar flexion contractions at 40, and 60% of MVC. The exercise protocol involved rhythmic isometric contractions in an equal work to rest ratio (2.5-s contraction: 2.5-s rest) for a total of 2 min. The pattern was maintained by following a digital metronome (total of 24 contractions). This exercise time was chosen as it elicited steady-state HHb in pilot trials. A feedback display of the actual force output was provided to the participants who matched it at the prescribed intensity. Recovery between the different intensity bouts was 3 min, or until NIRS data returned to baseline. Values for HHb and tHb were reported as the delta from baseline (30 s average prior to test) to steady state exercise to examine the metabolic demands of the exercise bout. The HHb signal which is regarded as blood volume insensitive during exercise was therefore used to indicate intramuscular oxygenation status (De Blasi et al., 1994). Following the cessation of exercise, the time course of recovery from exercise was modeled on O_2_Hb off-kinetics to evaluate oxygen delivery and utilization (McCully et al., 1994b). A mono-exponential (Eq. 4) was fitted to the first point greater than 1 SD above the end exercise mean over 120 s and a Tc calculated.

### NIRS Device

The NIRS signals were obtained using a portable unit consisting of 3 channels (Portamon, Artinis Medical Systems, Zetten, Netherlands). The system is a two-wavelength continuous wave system that simultaneously uses the modified Beer-Lambert law and spatially resolved spectroscopy methods. Changes in tissue O_2_Hb, HHb, and tHb were measured using the differences in absorption characteristics of infrared light at 760 and 850 nm. Differential path factor (DPF) of 4 was used throughout. NIRS data was connected to a computer by Bluetooth for acquisition at 10 Hz.

### IPC Protocol

For the IPC protocol, automatic inflation cuffs (14.5 cm width – Delfi Medical Innovations, Vancouver, BC, Canada) were placed on the proximal portion of both thighs. The inflatable cuffs were connected to a pressure gauge and were automatically inflated to 220 mmHg (IPC) to ensure maximum occlusion across all participants (de Groot et al., 2010). The sham group experienced a lower pressure (20 mmHg) using the same automatic inflation cuffs. The protocol involved 5-min occlusion, followed by 5-min reperfusion, which was repeated four times (lasting 40 min) in the supine position. This procedure was repeated for 7 consecutive days. To ensure the complete occlusion of arterial inflow to the limb, each individual had their limb occlusion pressure (LOP) assessed using a Doppler probe (UltraTec PD1, Ultrasound Technologies, Caldicot, United Kingdom) and automatic inflation cuff, as previously described (Loenneke et al., 2012). Average LOP for the IPC group was 140 ± 8 mmHg and for the sham group 151 ± 17 mmHg.

### ATT, Heart Rate, and Blood Pressure

Adipose tissue thickness (ATT) was measured at the site of the NIRS optode, in duplicate, to the nearest 0.1 mm using skinfold calipers (Harpenden, Burgess Hill, United Kingdom). The average value of skin and subcutaneous tissue thickness for the IPC group was 6.9 ± 2.6 mm and for the sham group 6.4 ± 2.5 mm (range of 3.8–12.4 mm). This was less than half the distance between source and the detector (35 mm). There was no correlation between m
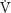
O_2_ and ATT when expressed as a percentage of the ischemic normalization (*R^2^* = 0.19437; *P* > 0.05). Blood pressure was monitored using an upper arm blood pressure monitor (Omron i-C10, Omron Healthcare Co., Kyoto, Japan) at the beginning of both experimental sessions, following 10 min of supine rest. Mean arterial pressure (MAP) was reported based on calculations of systolic blood pressure (SBP) and diastolic blood pressure (DBP): MAP = DBP + 0.33 (SBP - DBP). Following duplicate measures of oxidative capacity, heart rate responses were recorded in the supine position (Polar V800, Polar Electro OY, Kempele, Finland) for 2 min (pre-occlusion), 5 min (ischemic-normalization arterial occlusion), and 5 min (reperfusion). The periods were selected to obtain measurements of heart rate and heart rate variability (HRV) during rest and in response to acute lower-limb ischemia. The raw RR intervals were converted into beat-to-beat heart rate, after which potential ectopy and artifact was determined by a computer algorithm that identified all RR intervals outside the upper and lower limits (±25%) of a 5-beat moving average. Two time-domain measures of short-term HRV were determined: the standard deviation of all RR intervals (SDNN) and the square root of mean squared differences of successive normal RR intervals (RMSSD). Data were reported in milliseconds (ms). Conducting short-term HRV over = 2-min resting intervals has been commonly used in the literature (Dekker et al., 1997; Levy et al., 1998) as an indication of training adaptation or health status. The device used to measure HRV has been validated against ECG recordings (Giles et al., 2016). Room temperature and lighting was controlled throughout all trials (19.7 ± 0.5°C) and the participants were not given any instructions relating to their breathing patterns while measurements were taking place. The participants did not talk, consume food or drink, nor were they given any additional verbal or visual stimuli during the trial.

### Statistical Analysis

Data are presented as means ± SD. After checks for normality, analysis of covariance (ANCOVA) was used to determine the difference between resting m
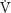
O_2_ and recovery kinetics, exercise intensity and recovery, heart rate, mean arterial pressure, and heart rate variability measurements. There was one independent variable with two levels (IPC vs. sham), with the participants’ pre-test baseline data used as a covariate. Magnitude of effects was calculated with partial eta-squared ($\eta_{p}^{2}$) according to the following criteria: 0.02, a small difference; 0.13, a moderate difference; 0.26, a large difference (Cohen, 1988). Statistical analysis was performed using SPSS 21 (IBM, Armonk, NY, United States). Curve fitting and display of data was performed with GraphPad Prism (GraphPad Software, La Jolla, CA, United States). Statistical significance was set at *P* < 0.05.

## Results

### Resting Muscle Oxygen Consumption

**FIGURE 3:**
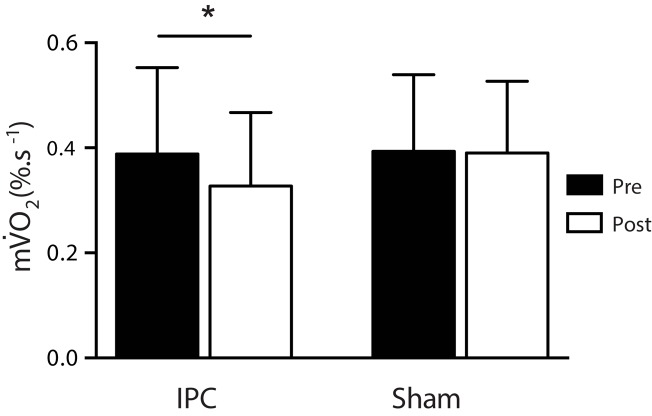
Resting muscle oxygen consumption pre (black) and post (white) for the IPC (*n* = 10) and sham (*n* = 10) groups. ^∗^*P* > 0.05.

Resting m
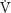
O_2_ was comparable at baseline between groups (IPC 0.39 ± 0.16%.s^-1^, Sham 0.39 ± 0.15%.s^-1^; *P* > 0.05). Following the 7-day IPC intervention, m
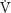
O_2_ was reduced by 16.4% (IPC 0.33 ± 0.14%.s^-1^) compared to no change in the control group (Sham 0.39 ± 0.14%.s^-1^) [*F*_(1,0.018)_ = 4.493, *P* = 0.039, $\eta_{p}^{2}$= 0.21], indicating a moderate effect (**Figure 3**).

### Muscle Oxidative Capacity

**FIGURE 4:**
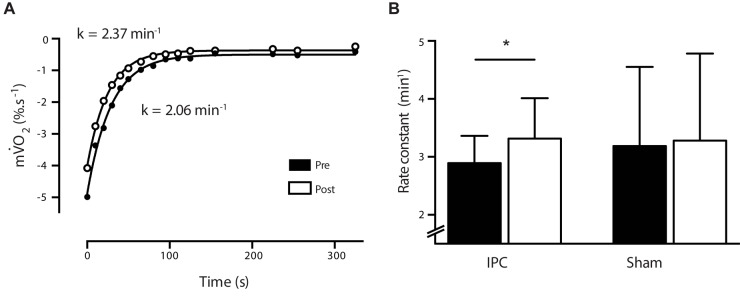
**(A)** Representative (*n* = 1) NIRS m
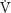
O_2_ recovery curve from pre (white circle) and post (black circle) following a 7-day IPC intervention. Data is fitted to a non-linear monoexponential curve and a rate constant (*k*) derived. **(B)** Bar graph illustrates average rate constants for pre (black) and post (white) for the IPC (*n* = 10) and sham (*n* = 10) groups. ^∗^*P* > 0.05.

Immediately following a 15-s electrical stimulation during pre-intervention testing, end contraction m
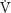
O_2_ was increased relative to resting m
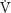
O_2_ in both conditions (IPC 0.39 ± 0.16 to 5.01 ± 0.92%.s^-1^; Sham 0.39 ± 0.15 to 4.09 ± 0.94%.s^-1^). Following the 7-day intervention procedure, there were no significant changes in end contraction m
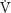
O_2_, however, $\eta_{p}^{2}$ indicated a moderate effect with a trend toward a ∼13% reduction in the IPC group (IPC pre 5.01 ± 0.92 to post 4.36 ± 0.38%.s^-1^) and minimal changes in the sham group (∼1.7%) (Sham pre 4.09 ± 0.94 to post 4.01 ± 0.68%.s^-1^) (*P* > 0.05, $\eta_{p}^{2}$ = 0.13). To model the recovery from 15-s electrically stimulated muscle contractions, the rate constant (*k*) for the recovery of m
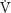
O_2_ was assessed via a series of brief occlusions prior to the intervention procedure (Baseline IPC 2.89 ± 0.47 min^-1^; Sham 3.18 ± 1.36 min^-1^). The 72 h following 7-day IPC, the rate constant for m
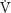
O_2_ recovery was increased ∼13%, with no change in the control group (< 3%) (IPC pre 2.89 ± 0.47 to post 3.32 ± 0.69 min^-1^; Sham pre 3.18 ± 1.36 to post 3.25 ± 1.50 min^-1^) [*F*_(1,19)_ = 4.897, *P* = 0.041, $\eta_{p}^{2}$ = 0.224], indicating a moderate effect (**Figures 4A,B**). A total of 9 out of the 10 participants in the IPC group demonstrated enhanced oxidative capacity (IPC range: 2–50%), with minor changes in the control group (Sham range: < 7%).

### Muscle Oxygenation Responses to Submaximal Exercise

**FIGURE 5:**
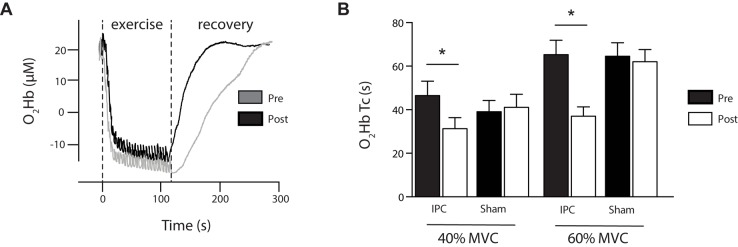
**(A)** Representative exercise protocol showing submaximal intermittent plantar flexion contractions at 40% 1RM and the subsequent reoxygenation in recovery. Data shown is from NIRS O_2_Hb and presented as pre (gray) and post (black) 7-day IPC. Note: recovery is faster (*P* < 0.05) following 7-day IPC. **(B)** The time constant (Tc) of recovery O_2_Hb following exercise at 40 and 60% MVC is presented for IPC and Sham as pre (black) post (white). ^∗^*P* > 0.05.

The IPC reduced delta HHb at exercise intensities of 40% MVC (IPC 16%; Sham 2% [*F*_(1,13)_ = 8.867; *P* = 0.014; $\eta_{p}^{2}$ = 0.470]) and 60% MVC (IPC 12%; Sham 3% [*F*_(1,13)_ = 4.217; *P* = 0.05; $\eta_{p}^{2}$ = 0.277]) with no change in sham (**Table 1**). In contrast, tHb was not different between condition (*P* > 0.05) (**Table 1**). The Tc for recovery following exercise was increased by ∼33 and 43% at 40 and 60% of MVC, respectively [40% MVC *F*_(1,13)_ = 0.824; *P* = 0.038; $\eta_{p}^{2}$ = 0.46] [60% MVC *F*_(1,13)_ = 6.742; *P* = 0.025; $\eta_{p}^{2}$ = 0.38], with no changes in the sham group (**Figure 5B**). A representative figure illustrates the shift in reoxygenation after exercise at 40% MVC following 7-day IPC (**Figure 5A**).

**Table 1:** Delta changes in HHb and tHb following submaximal intermittent plantar flexion contractions at 40 and 60% of 1RM among IPC (*n* = 7) and sham (*n* = 7) groups.

	(HHb) delta (a.u.)		(tHb) delta (a.u)	
	
	IPC Pre	IPC Post	IPC Pre	IPC Post
40% MVC	26.08 ± 5.81	21.96 ± 6.49^∗^	55.81 ± 8.77	54.39 ± 8.80
60% MVC	29.06 ± 7.59	25.62 ± 6.79^∗^	66.01 ± 7.68	64.60 ± 13.77
	
	**Sham Pre**	**Sham Post**	**Sham Pre**	**Sham Post**
	
40% MVC	25.62 ± 11.26	26.05 ± 6.96	43.96 ± 15.84	45.97 ± 8.87
60% MVC	25.88 ± 9.92	27.76 ± 9.53	50.08 ± 16.99	51.65 ± 9.76

*Values are presented as mean ± SD. HHb, deoxygenated hemoglobin; tHb, total hemoglobin; MVC, maximal voluntary contraction; a.u., auxillary units; ^∗^P < 0.05 comparing pre to post.*

### Cardiovascular Adaptations

Mean arterial pressure (MAP) was not different between groups (*P* = 0.578; $\eta_{p}^{2}$ = 0.019) (**Table 2**). There was no difference between IPC and sham groups for post-trial measures of 2-min resting heart rate (*P* = 0.255; $\eta_{p}^{2}$ = 0.085), 2-min RMSSD (*P* = 0.110; $\eta_{p}^{2}$ = 0.162), 2-min SDNN (*P* = 0.076; $\eta_{p}^{2}$ = 0.195), 5-min occlusion heart rate (*P* = 0.531; $\eta_{p}^{2}$ = 0.027), and 5-min recovery heart rate (*P* = 0.867; $\eta_{p}^{2}$ = 0.002) (**Table 2**).

**Table 2:** Heart rate, heart rate variability, and mean arterial pressure (MAP) during rest and occlusion among IPC (*n* = 10) and sham (*n* = 10) groups.

	Sham Pre	Sham Post	IPC Pre	IPC Post
2-min resting HR (b⋅min^-1^)	62 ± 9	62 ± 9	60 ± 8	64 ± 11
2-min RMSSD (ms)	145 ± 98	96 ± 19	169 ± 133	143 ± 107
2-min SDNN (ms)	113 ± 87	62 ± 19	139 ± 136	103 ± 74
5-min occlusion HR (b⋅min^-1^)	63 ± 8	63 ± 6	61 ± 9	63 ± 9
5-min post-occlusion HR (b⋅min^-1^)	61 ± 9	62 ± 7	60 ± 9	63 ± 10
MAP (mmHg)	89 ± 10	87 ± 6	89 ± 6	88 ± 6

*Values are expressed as mean ± SD. HR, heart rate; RMSSD, the square root of mean squared differences of successive normal RR intervals; SDNN, standard deviation of the normal RR intervals; MAP, mean arterial pressure.*

## Discussion

This study provides the first evidence that skeletal muscle oxidative function is enhanced following repeated IPC. We report a number of important observations. First, repeated bouts of bilateral lower-limb IPC across 7 consecutive days increased skeletal muscle oxidative capacity. Second, we observed a decrease in resting skeletal muscle metabolism. Third, there was evidence of enhanced microvascular oxygenated blood flow following IPC that facilitated a rapid recovery from submaximal exercise. Finally, systemic measures of resting blood pressure, heart rate and autonomic function did not change following IPC. Together, these findings suggest that local adaptations, distal to the IPC stimulus, potentiate oxidative function, and microvascular blood flow beyond the late phase (24–72 h) of protection, despite no apparent systemic cardiovascular changes.

### Enhanced Local Skeletal Muscle Oxidative Capacity

A ∼13% increase in skeletal muscle oxidative function was achieved in this study, by administering four bouts of arterial occlusion for 5 min with a time equivalent reperfusion, over a 7-day period. The participant group used for this investigation were healthy, young (average ∼26 years), active males (average engagement in exercise ∼4 h/week), with baseline measures of oxidative function that support a good level of physical fitness (∼2.89 min^-1^) when compared values reported elsewhere using this NIRS technique. For example, elite athletic populations (VO_2peak_ ∼74 ml.kg^-1^.min^-1^) exhibit faster oxidative recovery (∼3.2 min^-1^) when compared to sedentary individuals (VO_2peak_ ∼34 ml.kg^-1^.min^-1^) (∼1.7 min^-1^) (Brizendine et al., 2013). In contrast, patients that share characteristics of exercise intolerance and fatigue during low-intensity work often demonstrate a reduction in muscle oxidative capacity. This has been described for physical inactivity (Ryan et al., 2013b), immobilization (Krieger et al., 1980), aging (McCully et al., 1994a; Larsen et al., 2009, 2012), chronic disease (Southern et al., 2015), and motor complete spinal cord injury (Levy et al., 1993; McCully et al., 2011; Erickson et al., 2013), as well as a variety of metabolic disorders, such as obesity (Wells et al., 2008), type 2 diabetes (Mogensen et al., 2007), and mitochondrial myopathy (van Beekvelt et al., 1999).

Trainable differences in oxidative function have been described using NIRS, where following a 4-week period of endurance training, wrist flexor muscles increased their oxidative function by 64% (Ryan et al., 2013b). Shorter bouts of training across 7–14 days also potentiate markers of mitochondrial adaptation, that underlie changes oxidative potential (Egan et al., 2013). Therefore, we have observed that repeated IPC can stimulate adaptive changes in skeletal muscle oxidative function that are replicate to changes initiated by exercise. Despite the moderate shift in oxidative capacity reported, the smaller margin for improvement in a sample population that is already healthy and trained, alongside the well-conditioned nature of the locomotive muscle groups examined, when compared to greater changes reported for relatively untrained wrist flexor muscles (Ryan et al., 2013b), should be noted. We would speculate that the potential for improvement in muscle oxidative capacity in the lower limbs could be much greater and more easily achieved in a sedentary or reduced mobility patient population and, thus clinically significant where exercise programs are not appropriate. While there has been limited research into the effects of repeated IPC, cardiovascular adaptations have been described when IPC is applied for 7 consecutive days (Jones et al., 2014), episodically across a four (Kimura et al., 2007), and 8-week period (Jones et al., 2015), and even twice daily application for 300 days (Meng et al., 2012). Therefore, the importance of repeated IPC as a simple, non-invasive clinical alternative to exercise prescription to enhance skeletal muscle oxidative function alongside its reported cardiovascular benefits, warrants further study.

### Resting Muscle Metabolism

In addition to enhanced oxidative capacity, a corresponding ∼16% decrease in resting muscle metabolism occurred following IPC. A similar observation has been made in porcine models, where skeletal muscle energy demand was reduced following preconditioning (Pang et al., 1995). We extend this to report a sustained reduction 72 h following the final bout of IPC. This may be explained by two potential mechanisms; either a reduction in metabolic rate (reduced ATP requirement) or an increased mitochondrial efficiency (increased ATP per molecule O_2_). Interestingly, no change in resting m
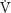
O_2_ was reported following 4 weeks of endurance exercise training of the wrist flexor muscles (Ryan et al., 2013b). However, in clinical populations, a decreased resting muscle m
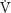
O_2_ has been reported in patients with chronic heart failure (Abozguia et al., 2008) and patients with severe peripheral arterial disease (Malagoni et al., 2010). In these cases, chronic reductions in oxygen supply to the muscle lead to adaptive changes in the periphery, which downregulate resting m
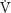
O_2_. Patients with mitochondrial myopathies also present with decreased resting m
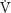
O_2_, in this case due to diminished oxidative function, which is compensated by increased blood flow to the muscle (van Beekvelt et al., 1999). In contrast, patients with reduced mobility, without impaired blood flow (i.e., multiple sclerosis patients), have an increased resting m
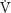
O_2_ (Malagoni et al., 2013). These peripheral adaptations occur to facilitate oxidative function, thus sustaining physical activity in these patients. Based on these observations, we would speculate that the enhanced oxidative function we report may enable a reduction in resting muscle metabolism via an increased mitochondrial efficiency.

Increases in VEGF and NO (Kimura et al., 2007) that have been reported following repeated IPC suggest that changes in oxidative capacity and resting metabolic rate could be linked to adaptations in mitochondrial function. During a bout of arterial occlusion, local tissue hypoxia occurs downstream of the occlusion site. Hypoxia stimulates VEGF, which has been shown to activate Akt3, ultimately facilitating nuclear localization of PGC-1α, the master regulator of biogenesis (Wright et al., 2008). In this case, it could be hypoxia *per se* that elicits an enhanced mitochondrial capacity, thereby improving oxidative function, both at rest and following exercise. An alternate explanation is that repeated bouts of ischemic exercise, independent of systemic hypoxia, augment mRNA content of PGC-1α, and markers of oxidative stress (Christiansen et al., unpublished). Through generation of reactive oxygen species (ROS) and subsequent activation of AMPK pathways, mitochondrial function is augmented. However, it important to note that excessive ROS production leads to oxidative stress, which has been implicated in the pathophysiology of cardiovascular diseases. Therefore, future work will need to establish the pathway by which augmented oxidative function is supported. A future consideration is the emerging role of post-translational modifications of mitochondrial proteins involved in oxidative phosphorylation and how hypoxic and ischemic stress may modulate their function (Hofer and Wenz, 2014).

### Vascular Adaptations Following IPC

We extended our primary hypothesis to speculate that if we found an improvement in oxidative function following repeated IPC, then this would translate to the exercising muscle. Acute application of IPC improves muscular endurance as evidenced by accelerated muscle deoxygenation kinetics (Kido et al., 2015; Tanaka et al., 2016). NIRS has also been used to describe the amplitude of response of HHb and tHb during exercise. The HHb signal, which is regarded as blood volume insensitive, is reported to reflect the balance between local oxygen delivery and utilization, meaning it can report muscle fractional oxygen extraction during exercise (De Blasi et al., 1993; Grassi et al., 2003). During intermittent isometric contractions, HHb was significantly reduced by ∼11–15% at differing exercise intensities following IPC. There are various interpretations of this finding such as a reduction in capillary recruitment during the exercise bout, a change in capillary-venous heme concentration, a reduction in muscle oxygen consumption, greater oxygen delivery, or a combination of these factors. That tHb remained unchanged, may suggest that oxygen delivery was increased following IPC via a vascular or vasodilatory mechanism.

The recovery of muscle oxygenation during a non-occluded state following exercise has also been used to evaluate oxidative function (Chance et al., 1992; McCully et al., 1994b). A delay in recovery following exercise is due to both the time course required for PCr resynthesis, provided that muscle pH remains relatively stable, and the delivery of oxygen (Chance et al., 1992; McCully et al., 1994b). During recovery from exercise, the balance between oxygen supply and oxygen demand shifts in favor of oxygen supply as bioenergetic resources are restored (Chance et al., 1992). In patients with peripheral vascular disease, oxygen resaturation is lengthened in the lower limb due to an inhibition of blood flow (McCully et al., 1994a, 1997). Therefore, the faster reoxygenation kinetics noted in the IPC group following exercise at both intensities (∼32–43%), may reflect first, an enhanced oxidative capacity and, second, increased oxygen delivery via a vasodilatory mechanism or vascular adaptation. Our data supports previous findings where simultaneous increases endothelial–dependent vasodilation (∼9%) and vascular conductance (∼14%) have been described following daily exposure to IPC for 7 days (Jones et al., 2014). Indeed, similar observations have also been made following intermittent exposure over 4 weeks (Kimura et al., 2007) and 8 weeks (Jones et al., 2015). The primary mechanism that has been proposed to mediate such changes has identified the sheer stress exerted on the blood vessels following a period of ischemia and subsequent reperfusion, as a major stimulus for microvascular adaptation and endothelial function due to the subsequent increase in blood flow following reperfusion (Green et al., 2010; Tinken et al., 2010). Interestingly, Jones et al. (2014) also reported vasculature adaptations in remote areas that were not directly exposed to the repeated ischemic stimuli, suggesting that circulating factors such as VEGF or endothelial progenitor cells (Kimura et al., 2007) may elicit both local and systemic effects. Much work remains to be carried out to understand the effect of IPC on local and systemic vascular and oxidative function.

### Systemic Cardiovascular Effects of IPC

Reductions in systolic and diastolic blood pressure have been observed following repeated IPC (Madias, 2011; Jones et al., 2014). However, we found no changes in blood pressure. There are inconsistencies in the literature, with several studies reporting no changes in blood pressure (Kimura et al., 2007; Madias, 2015) suggesting that more evidence is needed to understand the systemic effects of IPC. In addition, no changes in resting heart rate or autonomic function were noted between groups, as indicated by measurements of HRV. This was unanticipated, given the systemic effects of IPC on parasympathetic nervous system activity that have been demonstrated (Enko et al., 2011). Systemic cardiovascular protective effects of acute IPC are temporal in nature, with an early (1–4 h) and late (24–72 h) protective window (Yellon and Baxter, 1995). We postulate that the testing protocol utilized, whereby observations were made 72 h following the final bout of IPC, could also have permitted a partial decay in systemic cardiovascular and hemodynamic markers described. In addition, the cohorts studied were healthy, active, and already well-conditioned to exercise, which may have reduced the potential for changes in cardiovascular function. It would appear that the nature and time-course of the applied stimulus may need to be examined in more detail. It may be that systemic effects occur acutely after IPC or that repeated daily frequency is needed for these improvements to be sustained.

### Risks Associated With IPC

Prolonged ischemia and reperfusion generates local tissue damage and can lead to endothelial injury, which may further impede vascular blood flow (Thijssen et al., 2016). While the implementation of IPC using a similar 7-day protocol prior to an ischemia-reperfusion injury has been shown to be effective in protecting against endothelial dysfunction (Luca et al., 2013), the question remains as to what is regarded as a safe dose. In addition, IPC has been shown to increase pro-inflammatory markers of leukocyte activation which could be of potential risk to cardiac patients (Zaugg and Lucchinetti, 2015). Therefore, the long-term safety and optimal dose of IPC remains a real consideration in clinical settings where underlying vascular issues could be compounded. Based on current evidence, when administered as a series of brief occlusions, little known side effects have been reported for a single application, nor for repeated applications up to 300 days (Meng et al., 2012). IPC also remains an effective technique to elicit cardioprotection in patients undergoing cardiac surgery.

### Limitations

A potential confounding factor in this study was the control over the level of exercise fitness in the individuals tested. This may have impacted the ability to enhance skeletal muscle function using IPC. We reported on average 4 h/week exercise that consisted of running or team sport training. However, further insight into the effectiveness of IPC to enhance skeletal muscle oxidative function in athletic populations, sedentary and/or clinical populations with reduced muscle activity, is needed. For the IPC stimulus itself, the optimal strategy in terms of number of repeated applications required, length of occlusion, number of occlusions, and timings of protection to elicit these oxidative changes, as well as safety, presents future questions for the scientific community. In addition, the remote effects on skeletal muscle not directly exposed to the ischemic stimuli also warrant further study. Heterogeneity of blood flow under the NIRS optode may also have impacted measurements of skeletal muscle oxidative function. We attempted to minimize this by standardizing placement of the optode and maintaining standard laboratory conditions for all testing. NIRS signal penetration is estimated to be one-half of the interoptode distance (in this case 35 mm), therefore, we monitored participants to ensure that subcutaneous adipose tissue was > 15 mm to limit absorption and scattering of light. Additional studies using multiple-source detector probes would help further validate our findings. Finally, the contribution of myoglobin to the NIRS signal remains controversial, with suggestions ranging from 20% at rest to up to 70% during exercise (Marcinek et al., 2007; Davis and Barstow, 2013). Separation of these spectra cannot be performed using the NIRS device used in this study. Therefore, future work will need to discriminate whether these contributions affect the measures of oxidative function performed in this study.

## Conclusion

Skeletal muscle is highly adaptable to multiple physiological stimuli. Here, repeated bouts of IPC stimulus over 7 consecutive days increased skeletal muscle oxidative capacity, decreased resting muscle metabolism, and enhanced microvascular oxygenation. It is clear that the adaptations reported may have resulted from changes in hemodynamics induced by a single bout of IPC, activation of molecular pathways, or diffusible factors that mediate protective effects. Repeated activation of these mechanisms over 7 days could explain the long-term benefits beyond the reported acute late phase of protection following IPC (+ 72 h). Enhancement of skeletal muscle oxidative potential, described here, has not been previously reported following repeated IPC but may suggest a potential longer term effect on muscle performance. Future studies will need to address the mechanisms by which IPC enhances oxidative function at a cellular level, although it is likely that changes in mitochondrial function contribute to these effects. These findings will be of particular interest to athletic and clinical populations.

## Author Contributions

OJ, SP, and MW conceived and designed the research. OJ, SP, MW, and JP acquired, analyzed, and interpreted the data. OJ, SP, MW, and JP drafted, edited, and revised the manuscript. OJ, SP, MW, and JP approved the final version of manuscript. All authors agreed to be accountable for all aspects of the work.

## Conflict of Interest Statement

The authors declare that the research was conducted in the absence of any commercial or financial relationships that could be construed as a potential conflict of interest.
